# Diagnostic accuracy of ultrasonographic features in detecting thyroid cancer in the transition age: a meta-analysis

**DOI:** 10.1530/ETJ-22-0039

**Published:** 2022-05-05

**Authors:** Alessia Cozzolino, Tiziana Filardi, Ilaria Simonelli, Giorgio Grani, Camilla Virili, Ilaria Stramazzo, Maria Giulia Santaguida, Pietro Locantore, Massimo Maurici, Daniele Gianfrilli, Andrea M Isidori, Cosimo Durante, Carlotta Pozza, on behalf of TALENT Group

**Affiliations:** 1 Department of Experimental Medicine, Sapienza University of Rome, Rome, Italy; 2 Service of Medical Statistics and Information Technology, Fatebenefratelli Foundation for Health Research and Education, Rome, Italy; 3 Department of Biomedicine and Prevention, University of Rome Tor Vergata, Rome, Italy; 4 Department of Translational and Precision Medicine, Sapienza University of Rome, Rome, Italy; 5 Endocrinology Unit, Department of Medico-Surgical Sciences and Biotechnologies, ‘Sapienza’ University of Rome, Latina, Italy; 6 Endocrinology Unit, Fondazione Policlinico Universitario Agostino Gemelli-IRCCS, Università Cattolica del Sacro Cuore, Rome, Italy

**Keywords:** thyroid nodules, thyroid cancer, transition age, ultrasonography, fine needle aspiration

## Abstract

**Context:**

Significant uncertainty exists about the diagnostic accuracy of ultrasonographic (US) features used to predict the risk of thyroid cancer in the pediatric population. Moreover, there are no specific indications for thyroid nodule evaluation in patients during the transition age.

**Objective:**

The meta-analysis aimed to address the following question: which thyroid nodule US features have the highest accuracy in predicting malignancy in the transition age.

**Methods:**

We performed a meta-analysis of observational/cohort/diagnostic accuracy studies dealing with thyroid nodule sonography, reporting US features, and using histology as a reference standard for the diagnosis of malignancy and histology or cytology for the diagnosis of benignity in the transition age (mean/median age 12–21 years).

**Results:**

The inclusion criteria were met by 14 studies, published between 2005 and 2020, including 1306 thyroid nodules (mean size 17.9 mm) from 1168 subjects. The frequency of thyroid cancer was 36.6%. The US features with the highest diagnostic odds ratio (DOR) for malignancy were the presence of suspicious lymph nodes (DOR: 56.0 (95% CI: 26.0–119.0)), a ‘taller than wide’ shape of the nodule (6.0 (95% CI: 2.0–16.0)), the presence of microcalcifications (13.0 (95% CI: 6.0–29.0)) and irregular margins (9.0 (95% CI: 5.0–17.0)). Heterogeneity among the studies was substantial.

**Conclusions:**

Following the diagnosis of a thyroid nodule in the transition age, a thorough US examination of the neck is warranted. The detection of suspicious lymph nodes and/or thyroid nodules with a ‘taller than wide’ shape, microcalcifications, and irregular margins is associated with the highest risk of malignancy in the selection of nodules candidates for biopsy.

## Introduction

Thyroid nodular disease is less frequent in children than in adults. The prevalence of palpable nodules in the adult population is 4–7%, and when the detection is made by ultrasound (US) or autoptic exam, the percentage arise to 50% (1). Conversely, it has been reported that approximately 1–1.5% of children and about 10% of adolescents and young adults have thyroid nodules (2).

Hayashida* et al*, in a study including 4365 patients between 3 and 18 years, identified solid nodules with a maximum diameter >5 mm in 1.01% of the total population, with a significantly higher prevalence in older patients and in the female group (3). Noticeably, the cancer rate is significantly higher in pediatric thyroid nodules than in the adult ones, being about 25 and 7%, respectively (4). Moreover, in a retrospective study encompassing 170 young patients with differentiated thyroid carcinoma (3–21 years) a recurrence rate of 17% has been observed (5). Based on these findings, thyroid nodules in pediatric patients require a careful evaluation.

According to the 2015 American Thyroid Association (ATA) Guidelines on Pediatric Thyroid nodules and Differentiated Thyroid Cancer, the evaluation and treatment of thyroid nodules in children should be the same as in adults with a few exceptions. In particular, clinical context and US characteristics should be used rather than size to identify nodules that warrant fine-needle aspiration (FNA), because the use of nodule size as a discriminating criterion in children may not be feasible due to age-related changing in thyroid volume (4). As for the clinical context, several risk factors for developing thyroid nodules and cancer in children have been identified: iodine deficiency, autoimmune thyroid disease (e.g. Hashimoto’s thyroiditis), prior radiation exposure, as well as genetic syndromes (APC (associated polyposis conditions), Carney complex, DICER1 syndrome, PTEN hamartoma tumor syndrome, and Werner syndrome) (4).

Several observational studies, mostly retrospective, have been conducted to identify the US features associated with the risk of malignancy in pediatric thyroid nodules. In 2016, they have been summarized in a meta-analysis reporting that the presence of internal calcifications and enlarged cervical lymph nodes were the US features with the highest likelihood ratio for thyroid cancer, being a cystic composition suggestive of benign nodules (6). Furthermore, a recent meta-analysis evaluated the performance of adult based ATA and American College of Radiology (ACR) US risk stratification systems (RSSs) in the pediatric setting: a fairly modest diagnostic accuracy came out, as well as the need for an appropriate tune-up for those RSSs to be applicable to the pediatric population (7). The aforementioned meta-analyses included studies on both children and young adult patients, plotted together. It is worthy of note that there are no specific indications for thyroid nodule evaluation in patients belonging to the transition age, which is defined as the period between the end of puberty and the achievement of peak bone mass, in an age range between 12 and 21 years (8). Therefore, the current meta-analysis aimed to bridge this gap by addressing the following issue: which thyroid nodules US features have the highest accuracy in predicting malignancy in the transition age?

## Methods

The study was pre-registered at the International prospective register of systematic reviews (registration no: CRD42020164803). This manuscript is reported according to the Preferred Reporting Items for Systematic Reviews and Meta-analysis guidelines (9).

### Eligibility criteria and study selection

Observational/cohort/diagnostic accuracy studies dealing with thyroid nodule classification reporting US features in the transition age were selected. Inclusion criteria were (i) use of histology as a reference standard for the diagnosis of malignancy and histology or cytology as a reference standard for the diagnosis of benignity; (ii) mean/median age of patients included in the studies ranging from 12 to 21 years. Conference abstracts, review and editorial articles, and case reports were excluded.

### Search strategy

Keywords and MeSH terms were identified and searched in PubMed. Publication language was restricted to English. The search query was (“Adolescent”[tiab] OR “Adolescence”[tiab] OR “young adult”[tiab] OR “young adults”[tiab] OR pediatric[tiab] OR pediatrics[tiab] OR children[tiab] OR child[tiab] OR childhood[tiab]) AND (“Thyroid Neoplasms”[Mesh] OR “thyroid nodule”[tiab]). Reference lists of selected studies were searched to identify additional relevant publications.

### Data extraction

Two investigators independently screened the papers retrieved during the searches, by their titles and abstracts, to identify those that were potentially eligible. The full texts of these studies were then assessed against the inclusion criteria and selected or rejected as appropriate. Data were subsequently extracted in duplicate, using a standard spreadsheet.

The following information was extracted and collected: (i) general information on the study (author, year of publication, institution, country, study type and design, number of patients, number of eligible patients, population age, and distribution); (ii) applied reference standard (histology or cytology); (iii) rate of benign and malignant nodules; (iv) for each considered feature, the corresponding number of true negative, true positive, false negative, false positive.

### Risk of bias assessment

Data were cross-checked for accuracy and completeness, resolving discrepancies by consensus or by a third reviewer. The risk of bias of the included studies was assessed using the Quality Assessment of Diagnostic Accuracy Studies (QUADAS-2) tool (10).

### Statistical methods

Performance of ultrasound risk stratification systems and single sonographic features in the selection of thyroid nodules for FNA was summarized using pooled sensitivity, specificity, negative predictive value, positive predictive value, and diagnostic odds ratio (DOR). Meta-analysis of binary diagnostic test accuracy was performed by the bivariate mixed-effects regression model, making inferences about average sensitivity and specificity. Average sensitivity and specificity, the likelihood ratio (LR) for positive and negative test results, and odds ratios are calculated from the maximum likelihood estimates. To quantify the test performance, the areas under the curve were calculated. For all estimates, the corresponding 95% CI were reported. Heterogeneity was quantified by the Higgins I2, a value of 0% indicates no observed heterogeneity, and values greater than 50% may be considered substantial heterogeneity (11).

Univariable bivariate meta-regression model was performed to investigate heterogeneity assuming the reference test as covariate. The effect of covariate on sensitivity was estimated separately from that on specificity.

Testing for publication bias was conducted by a regression of diagnostic log odds ratio against 1/sqrt (effective sample size), weighting by effective sample size (12). A *P* value <0.10 for the slope coefficient indicated a significant asymmetry (11).

A subgroup for sensitivity analysis was performed considering only studies with a low risk of bias in QUADAS-2. All statistical analyses were performed by STATA using the *midas* program.

## Results

### Study selection

**Figure 1:**
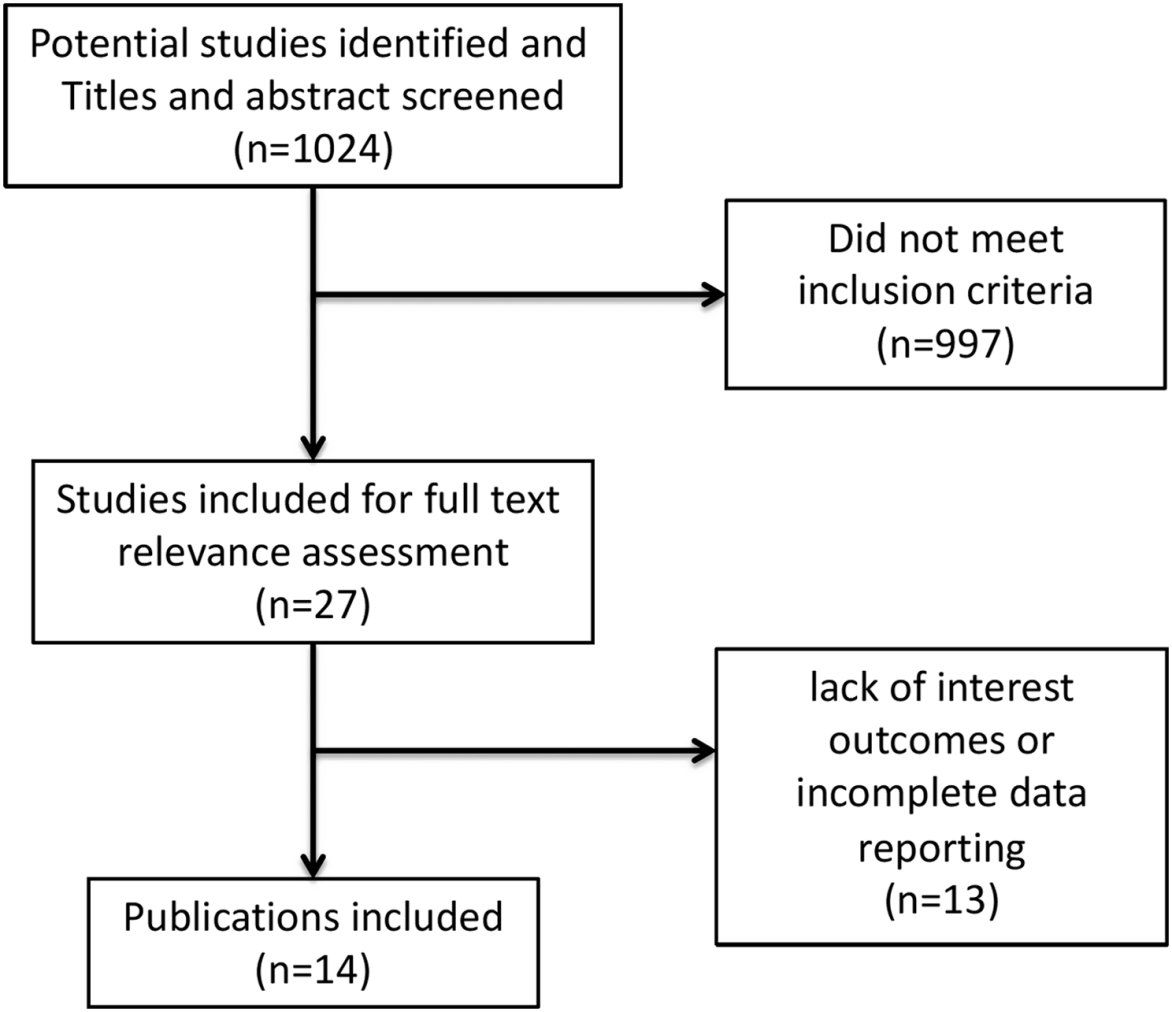
Flowchart of literature eligibility assessment process.

Figure 1 shows the literature eligibility assessment process. The data search identified 1024 potentially relevant studies, screened by title and abstract. Among these, 997 did not meet the inclusion criteria and were excluded. The main reasons for exclusion were the article type (reviews, case reports, and non-original study), the non-English language, and the lack of sonographic data in the study. This left 27 studies for full-text assessment, and 13 were excluded for the lack of interest outcomes or for incomplete data reporting. Ultimately, 14 studies were eligible to perform the meta-analysis (13, 14, 15, 16, 17, 18, 19, 20, 21, 22, 23, 24, 25, 26).

### Study characteristics

Table 1 summarizes the details of the 14 selected studies. All the included studies were cohort studies (2 prospective (20, 22) and 12 retrospective studies (13, 14, 15, 16, 17, 18, 19, 21, 23, 24, 25, 26)). Data were available from 1168 subjects, 194 males and 835 females (four studies did not specify sex) (15, 17, 24, 26), with a mean age of 14.6 years (range 2–21 years) and a total number of 1306 thyroid nodules. One study included only patients with a history of radiation exposure from the Chernobyl disaster (20). Among these 1306 nodules, 407 were found to be malignant based on the gold standard (histology), with an overall prevalence of thyroid cancer of 36.6%. The most common type of thyroid cancer was papillary thyroid cancer (92.1%) followed by follicular cancer (4.4%), medullary thyroid cancer (2.4%), and Hurtle cell carcinoma (1.1%).

**Table 1:** Details of selected studies.

Study name	Country	Objective of study	Study type	Reference standard	Number of patients (no of cases^a^)
Lyshchik 2005	Belarus	To prospectively analyze the accuracy of various diagnostic criteria for cancer in solid thyroid nodules in children on the basis of gray-scale and power Doppler ultrasonographic findings.	Prospective study	Histopathology or FNA with follow-up	103 (103)
Corrias 2008	Italy	To investigate the association between juvenile autoimmune thyroiditis (JAT) and thyroid cancer in pediatric patients	Retrospective study	Histopathology or FNA with follow-up	115 (48)
Roy 2011	USA	To investigate clinical factors that may predict malignancy in pediatric thyroid nodules	Retrospective study	Histopathology	207 (72)
Saavedra 2011	Canada	To assess whether the presence of criteria for malignancy on the initial thyroid ultrasonography was helpful in diagnosing thyroid cancer even when a fine-needle aspiration biopsy (FNAB) suggests a benign lesion	Retrospective study	Histopathology	35 (21)
Goldfarb 2012	USA	To determine whether the preoperative clinic-based ultrasound (CBUS) characteristics of pediatric thyroid nodules were able to help further guide management and treatment	Retrospective study	Histopathology	50 (50)
Mussa 2015	Italy	To evaluate the diagnostic accuracy of clinical, laboratory, and ultrasound imaging characteristics of thyroid nodules in assessing the likelihood of malignancy	Retrospective study	Histopathology or FNA with follow-up	184 (129)
Papendieck 2015	Argentina	To highlight the findings of each diagnostic tool likely to differentiate benign from malignant thyroid nodules in a large cohort of pediatric patients	Prospective study	Histopathology or FNA with follow-up	75 (75)
Canfarotta 2017	USA	To evaluate the clinical utility of a modified pediatric McGill Thyroid Nodule Score (MTNS) with children and adolescents	Retrospective review	Histopathology	46 (46)
Lim-Dunham 2017	USA	To evaluate the diagnostic performance of pediatric thyroid nodule risk stratification for predicting malignancy when applying the ultrasound criteria recommended	Retrospective study	Histopathology or FNA with follow-up	33 (33)
Hammond 2017	USA	To evaluate the risk of thyroid cancer in incidental thyroid nodules discovered on CT in patients with a history of pediatric cancer	Retrospective review	Histopathology	20 (6)
Richman 2018	USA	To determine the relationship between demographic and sonographic characteristics of thyroid nodules and malignancy in a pediatric population	Retrospective study	Histopathology or FNA with follow-up	314 (314)
Uner 2019	Turkey	To define the diagnostic power of the TI-RADS risk stratification method in pediatric thyroid nodules.	Retrospective study	Histopathology or FNA with follow-up	64 (64)
Lim-Dunham 2019	USA	To assess the diagnostic performance of the American College of Radiology (ACR) Thyroid Imaging Reporting and Data System (TIRADS) for malignancy risk in pediatric thyroid nodules	Retrospective study	Histopathology or FNA with follow-up	62 (62)
Suh 2020	Korea	To identify predictive factors of thyroid cancer	Retrospective study	Histopathology or FNA with follow-up	275 (145)

^a^Number of patients refers to the whole population included in the study, whereas number of cases refers to the patients finally included in the analysis.

All the included studies reported thyroid US features. Only four studies referred to US scores: in particular, two studies used the ATA score (14, 18) and two studies used the ACR Thyroid Imaging Reporting and Data System score (19, 26).

The most frequently reported US features were: echogenicity in twelve studies (13, 14, 15, 16, 17, 18, 20, 21, 22, 23, 24, 25); margins in ten studies (13, 16, 17, 18, 19, 20, 21, 22, 24, 25); the presence of microcalcifications in eleven studies (13, 14, 16, 17, 18, 19, 20, 21, 22, 24, 25); ‘taller than wide’ shape in seven studies (13, 14, 16, 17, 18, 19, 24); vascularization in eight studies (13, 14, 18, 20, 21, 22, 24, 25); the presence of suspicious lymph nodes in seven studies (13, 14, 16, 18, 21, 22, 25). Only three studies evaluated the prognostic value of the US score used (18, 19, 26).

As per inclusion criteria, all the included studies used histology as a reference standard for the diagnosis of malignancy. Conversely, only six studies used histology as a reference standard for the diagnosis of benignity (14, 16, 17, 23, 24, 25), two studies used cytology (13, 22), and the remaining six studies used both cytology and histology (15, 18, 19, 20, 21, 26).

### Meta-analysis

**Figure 2:**
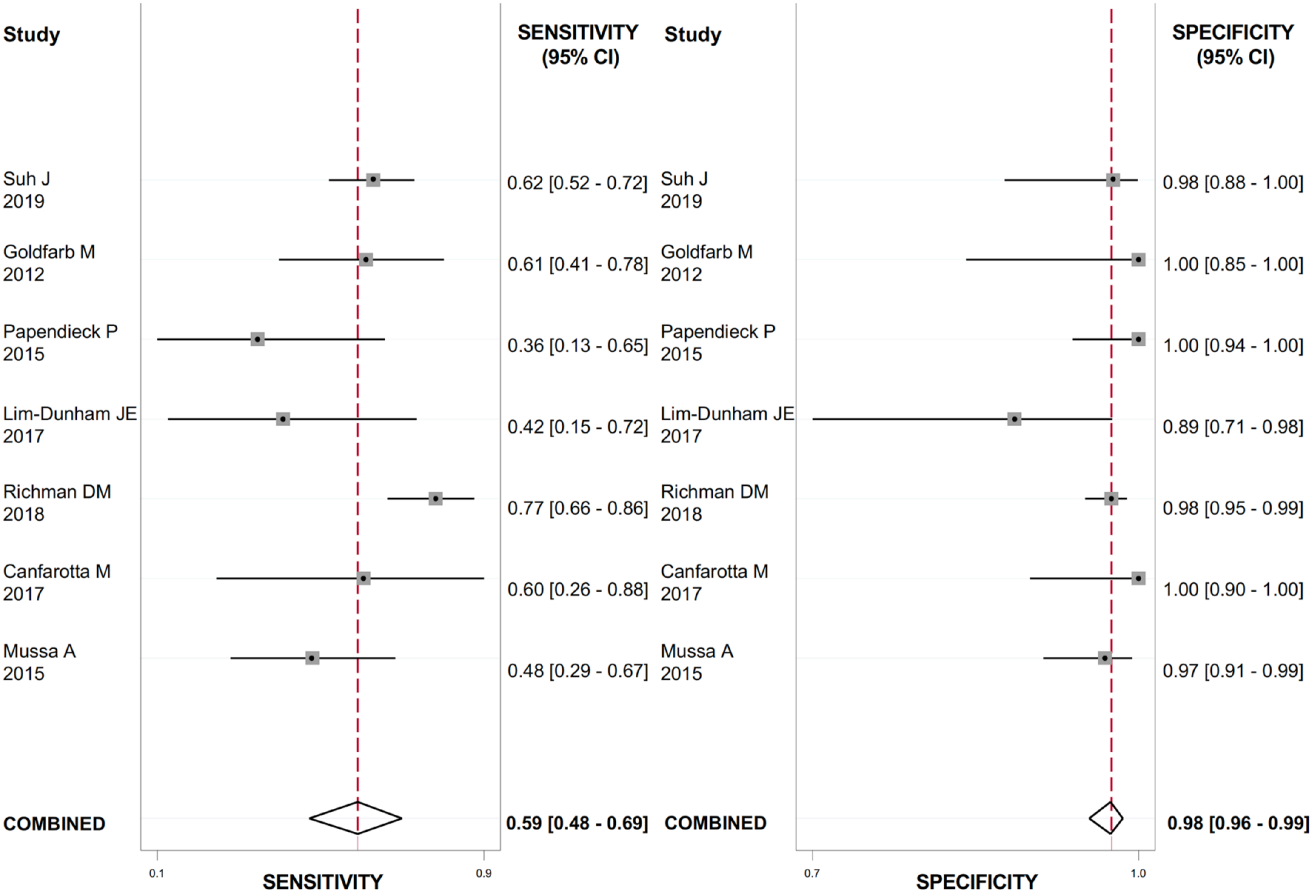
Forest plot of sensitivity and specificity estimates of diagnostic accuracy of suspicious lymph nodes in predicting malignancy. Single studies are identified by first authors and publication year.

**Figure 3:**
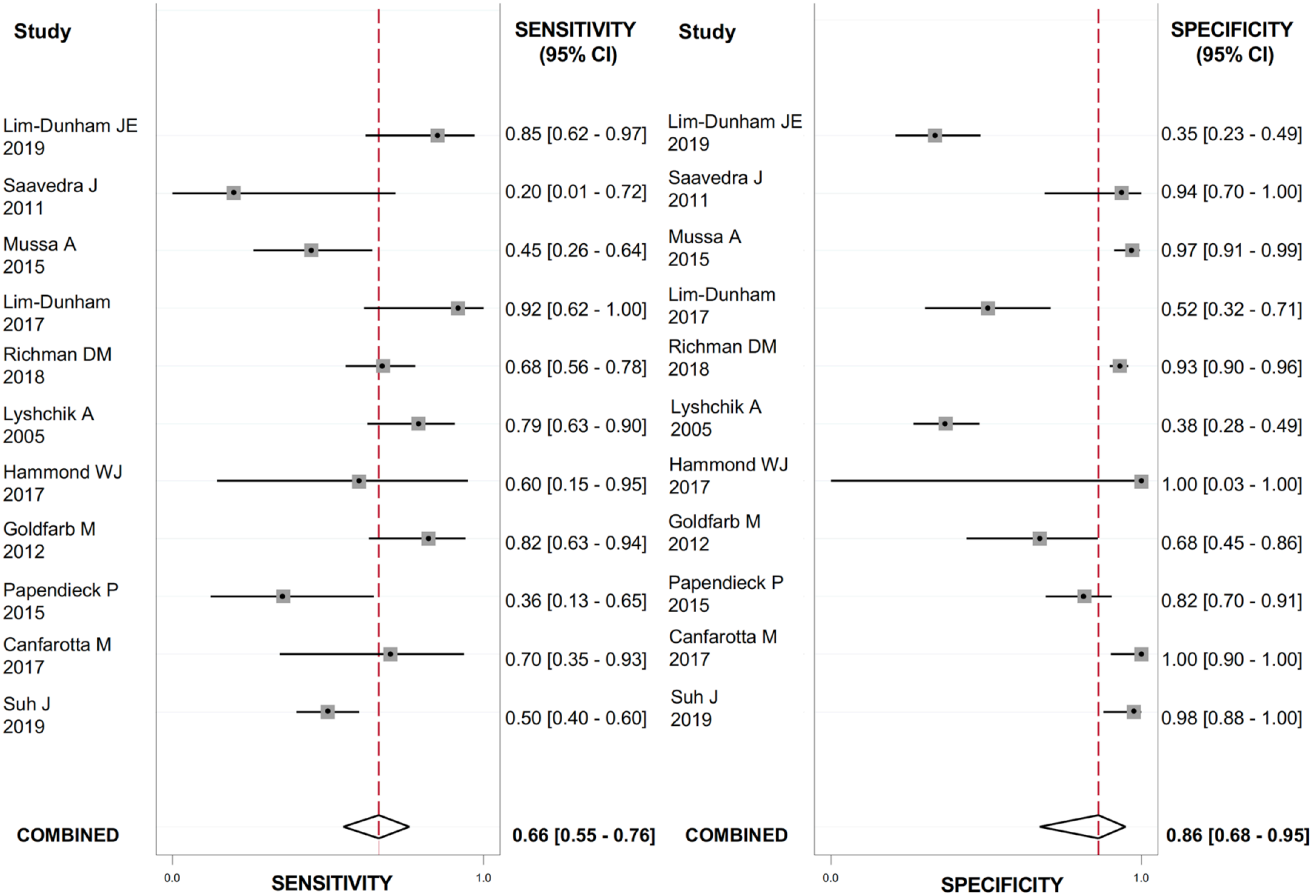
Forest plot of sensitivity and specificity estimates of diagnostic accuracy of microcalcifications in predicting malignancy. Single studies are identified by first authors and publication year.

**Figure 4:**
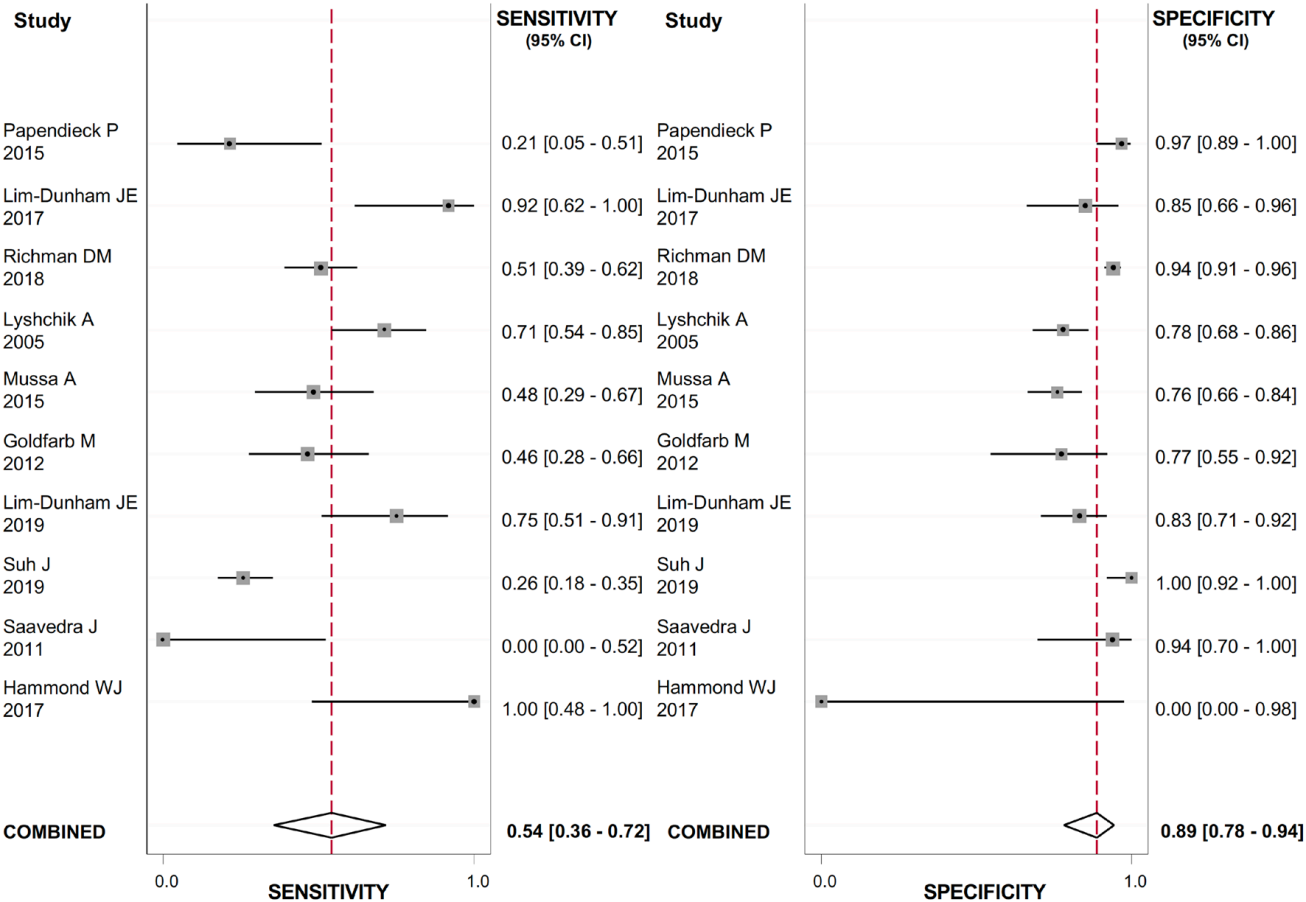
Forest plot of sensitivity and specificity estimates of diagnostic accuracy of irregular margins in predicting malignancy. Single studies are identified by first authors and publication year.

**Figure 5:**
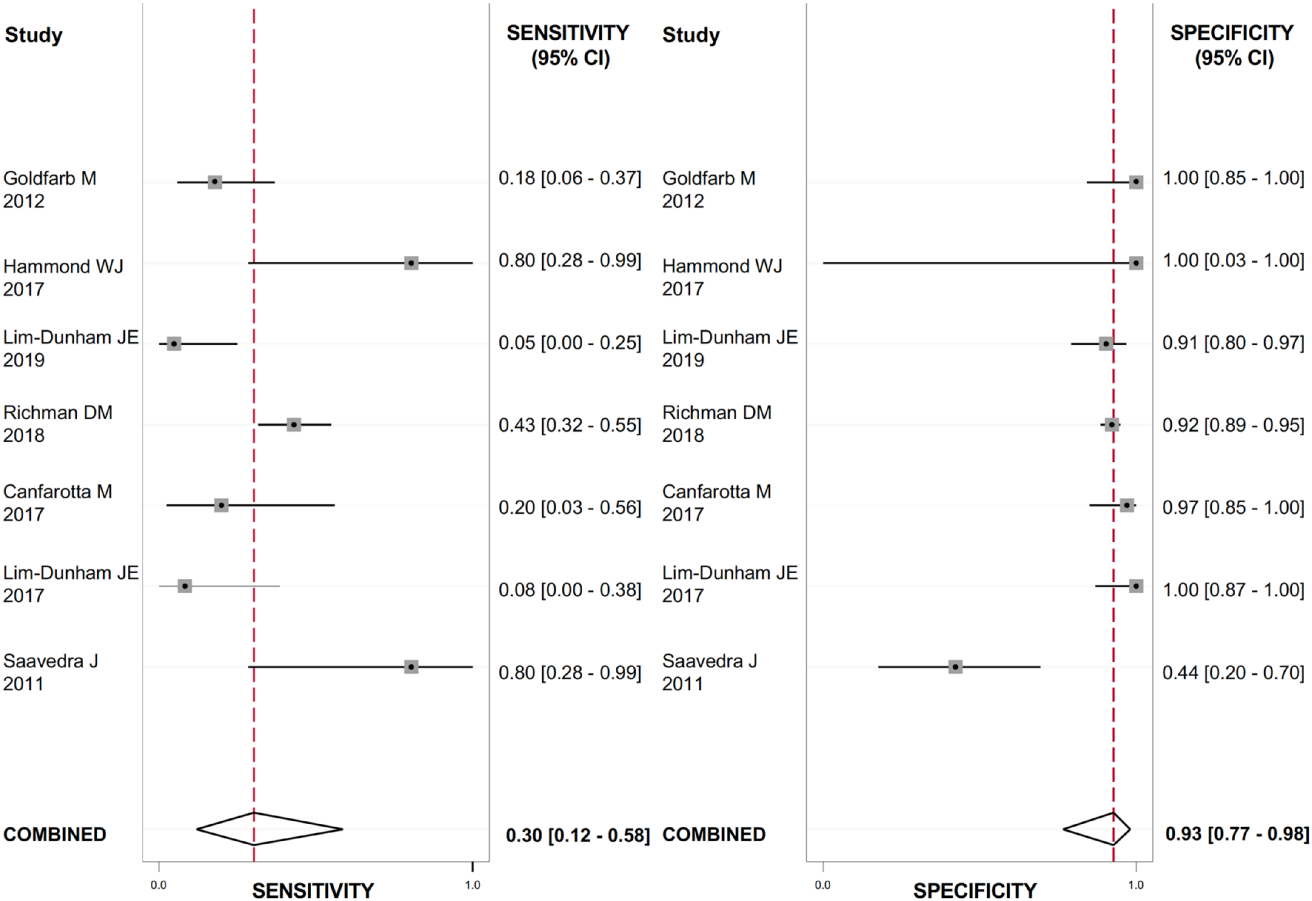
Forest plot of sensitivity and specificity estimates of diagnostic accuracy of ‘taller than wide’ shape in predicting malignancy. Single studies are identified by first authors and publication year.

The US features with the highest positive LR (LR+) for detecting thyroid cancer were the presence of suspicious lymph nodes (Fig. 2), evaluated in 888 nodules (LR+: 23.7; 95% CI: 12.8–43.9); the presence of microcalcifications (Fig. 3), evaluated in 1118 nodules (LR+: 4.9; 95% CI: 2.1–11.4); irregular margins (Fig. 4), evaluated in 1072 nodules (LR+: 4.8; 95% CI: 2.9–7.9); ‘taller than wide’ shape (Fig. 5), evaluated in 640 nodules (LR+: 4.3; 95% CI: 1.7–10.7).

These features had the highest DOR for thyroid cancer as well: 56.0 (95% CI: 26.0–119.0) for the presence of suspicious lymph nodes; 6.0 (95% CI: 2.0–16.0) for ‘taller than wide’ shape; 13.0 (95% CI: 6.0–29.0) for the presence of microcalcifications; 9.0 (95% CI: 5.0–17.0) for irregular margins. The heterogeneity between studies was substantial for all the US features evaluated (I^2^ ranged from 90 to 99%) except from suspicious lymph nodes, where no heterogeneity was observed. The results are reported in Table 2.

**Table 2:** Meta-analysis of binary diagnostic test accuracy of US features.

	Eco score	Ecogenicity	Ecotexture	Margins	Shape	Microcalcifications	Vascularization	Suspicious lymph nodes
*n* of study	3	12	8	10	7	11	8	7
*n* of nodules	181	1163	893	1072	640	1118	988	888
Sensibility	91.9% (61–98.8%)	58% (46–70%)	76% (26–97%)	54% (36–72%)	30% (12–58%)	66% (55–76%)	52% (25–78%)	59% (48–69%)
Specificity	51.8% (18.6–83.4%)	66% (56–74%)	71% (42–89%)	89% (78–94%)	93% (77–98%)	86% (68–95%)	65% (38–85%)	98% (96–99%)
LR+	1.9 (0.9–3.8)	1.7 (1.3–2.2)	2.6 (1.6–4.4)	4.8 (2.9–7.9)	4.3 (1.7–10.7)	4.9 (2.1–11.4)	1.5 (0.8–2.6)	23.7 (12.8–43.9)
LR-	0.16 (0.04–0.5)	0.63 (0.49–0.82)	0.34 (0.08–1.40)	0.52 (0.36–0.74)	0.75 (0.56–1.01)	0.39 (0.31–0.49)	0.74 (0.47–1.18)	0.42 (0.33–0.55)
DOR	12.75 (4.57–35.59)	3 (2–4)	8 (2–32)	9 (5–17)	6 (2–16)	13 (6–29)	2 (1–5)	56 (26–119)
AUC	83% (52–92%)	66% (62–70%)	79% (75–82%)	82% (78–85%)	74% (70–78%)	78% (74–81%)	61% (57–66%)	98% (96–99%)
I^2^	94.1%	94%	99.00%	96.00%	93.00%	99%	98%	0%
P	<0.001	<0.001	<0.001	<0.001	<0.001	<0.001	<0.001	0.26
Pub bias	*P* = 0.712	*P* = 0.096	*P* = 0.242	*P* = 0.282	*P* = 0.605	*P* = 0.356	*P* = 0.606	*P* = 0.137

LR+, the positive likelihood ratio; LR−, the negative likelihood ratio; DOR, diagnostic odds ratio; I^2^, heterogeneity among the studies.

### Risk of bias

**Figure 6:**
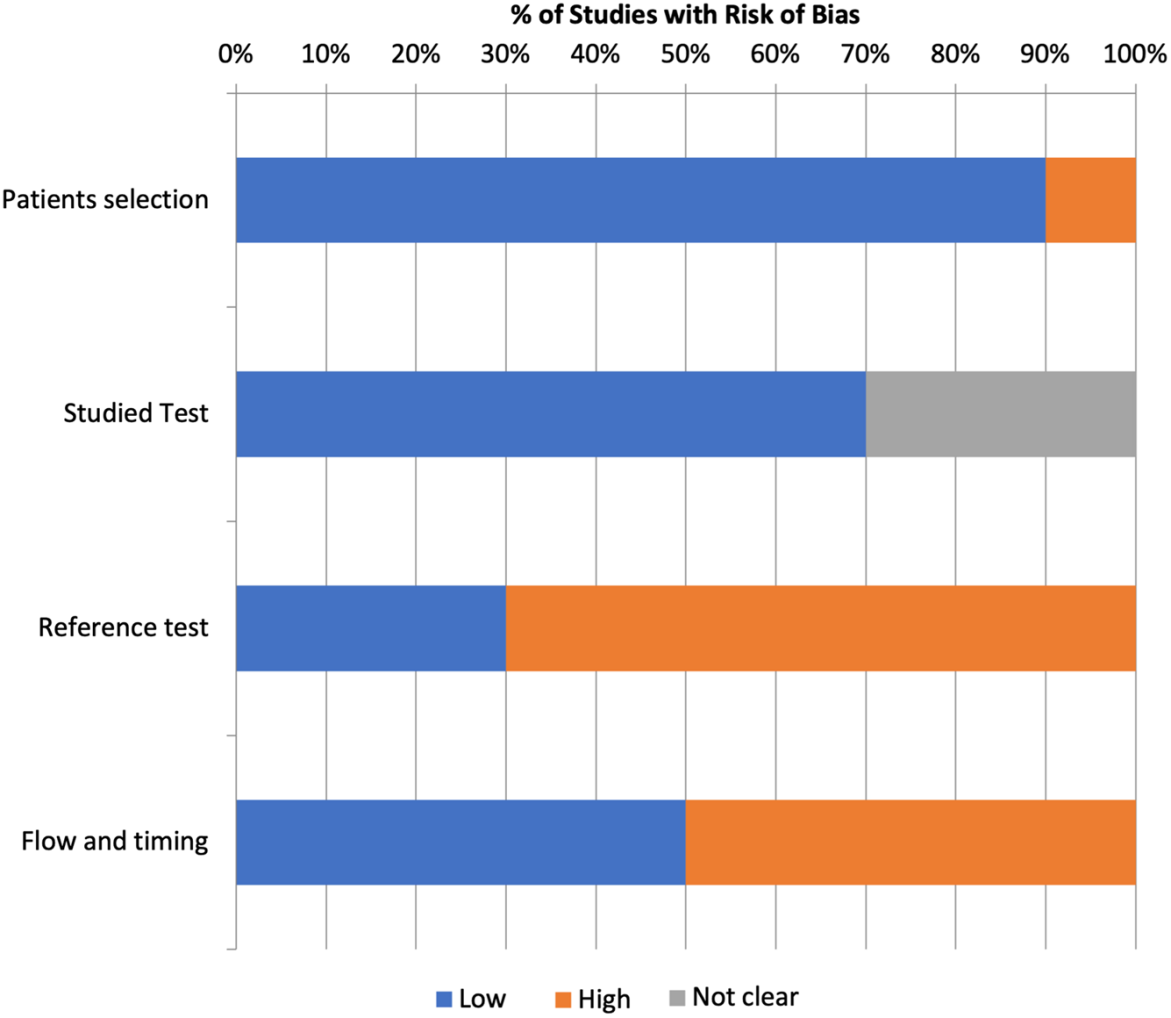
Risk of bias assessments.

The overall risk of bias was considered moderate. The most relevant methodological concerns related to the reference standard, since most of the included studies (eight studies) (13, 15, 18, 19, 20, 21, 22, 26) used cytology as reference standard for the diagnosis of benignity, determining a high risk of bias. The quality assessment using QUADAS-2 tool is summarized in Fig. 6.

Two subgroup sensitivity analyses were performed: (i) excluding the study including only patients exposed to Chernobyl disaster (20) and (ii) considering only studies with a low risk of bias according to QUADAS. The findings were consistent with the results of the meta-analysis considering all the studies. Specifically, the first subgroup analysis confirmed that the presence of microcalcifications, evaluated in 990 nodules (LR+: 6; 95% CI: 2.5–14) and irregular margins, evaluated in 943 nodules (LR+: 5.1; 95% CI: 2.8–9.1) were the US features with the highest LR+ for detecting thyroid cancer. Similarly, the results of the second subgroup analysis found that the presence of microcalcifications, evaluated in 984 nodules (LR+: 5.8; 95% CI: 2.4–13.7), irregular margins, evaluated in 937 nodules (LR+: 5.4; 95% CI: 3.0–9.8) and ‘taller than wide’ shape, evaluated in 634 nodules (LR+: 4.3; 95% CI: 1.5–12.6) had the highest diagnostic accuracy in detecting thyroid cancer.

## Discussion

This meta-analysis reveals that the identification at neck US of suspicious lymph nodes and/or thyroid nodules with a ‘taller than wide’ shape, microcalcifications, and irregular margins is associated with the highest diagnostic accuracy in detecting thyroid cancer in transition-age youths.

From a clinical perspective, thyroid nodules are less common among children than adults. However, nodules diagnosed in children carry a greater risk of malignancy and tend to present at a more advanced stage than in adults, with a higher frequency of lymph node metastases. The 2015 ATA guidelines for children with thyroid nodules (4) indicate that the evaluation and treatment of thyroid nodules in children should be the same as in adults, where FNA is not deserved if the nodule is smaller than 1 cm and there are no associated risk factors. However, a size criterion is not feasible in children since thyroid volume changes with age and nodule size alone cannot predict malignant histology. In the absence of accurate US predictors of malignancy, most of the nodules will require FNA, which carries its own set of costs and diagnostic challenges (27).

Many efforts have been made to improve the diagnostic work-up of thyroid nodules in the adult population and the most commonly used US RSSs have been demonstrated to allow high-confidence exclusion of malignancy in the assessment of thyroid nodules (28, 29), being particularly important in case of cytologically indeterminate ones (30, 31).

Moreover, most of the papers in the literature evaluating US features associated with a high risk of malignancy in pediatric thyroid nodules include both children and young adult patients, plotted together (6, 7). Therefore, there are no specific indications for thyroid nodule evaluation in patients belonging to the transition age.

The current meta-analysis included 14 studies, with a mean age of 14.6 years (range 2–21 years) and a total number of 1306 thyroid nodules. Based on the gold standard (histology), the prevalence of thyroid cancer was found to be 36.6%, slightly higher than that described in the literature (4). As expected, the most common type of thyroid cancer was papillary (92.1%) followed by follicular (4.4%), medullary (2.4%), and Hurtle cell carcinoma (1.1%). These results were substantially superimposable with the previous meta-analysis in the pediatric population (6).

In this meta-analysis, we have reported the probability of having a malignant tumor vs having a benign one in the transition age based on the presence of each feature and their LR. Tests with a low LR for negative results might rule out the risk of malignancy and the need for FNA, whereas tests with high LR for positive results might rule in the risk of malignancy and the need for FNA. This approach was applied to a population aged between 12 and 21 years. The results of this meta-analysis suggest that in transition-age high-risk features for thyroid malignancy are the presence of suspicious lymph nodes and/or nodules with a ‘taller than wide’ shape, microcalcifications, and irregular margins. Due to the small number of studies considering the most used US RSSs, we could not perform an analysis to measure their diagnostic accuracy in the transition age population.

Therefore, our findings could support the physician facing a thyroid nodule in the transition-age youth to choose whether further diagnostic tests are needed based on its US features. Specifically, they suggest that every patient in transition age with a thyroid nodule harboring one of the identified US features associated with a higher diagnostic OR for malignancy should undergo additional diagnostic evaluation, namely FNA and, conversely, if none of the aforementioned US features is present, the physician could adopt a conservative approach, for example, US follow-up.

A previous meta-analysis including 12 studies (6) suggested that a single thyroid US feature is not a highly accurate predictor of the nature of a thyroid nodule. Nevertheless, the authors found that internal calcifications, the presence of suspicious lymph nodes, irregular margins, and a solid echotexture were the features with the highest accuracy to detect thyroid cancer in children, and this is consistent with our results.

The current meta-analysis adds some significant novelties: first of all, the great majority of the studies included (85.7%) are after 2009, the time of the first proposal of a US RSS, with all investigations, from then on, being reporting the cardinal features aimed at assessing the thyroid nodule risk; it includes only studies using post-operative histology as a reference standard for malignancy, overpassing the bias of indeterminate cytology; finally, it investigates for the first time the diagnostic accuracy of US features in detecting thyroid cancer in the transition age.

However, it does have some limitations. Considering the relatively limited number of prospective studies involving transition-age patients, the current meta-analysis included mainly retrospective evidence. Large-scale prospective studies are therefore needed to draw firm conclusions. Another limitation is the substantial heterogeneity among the studies, although this is partially reduced by subgroup and sensitivity analyses. Furthermore, one of the included studies contributed over 30% of the examined nodules (13). Although the risk of bias in each study was examined and our results were adjusted, the effect of this study on the overall results remains to be considered. Of note, the detection of thyroid nodules characteristics might be influenced by US machine and US probes properties. Best identification of US thyroid nodules features requires high-quality ultrasound machines and an expert physician in interpreting the images (27). In addition, it was unknown if the evaluation of the US features was performed using real-time or static US images. Real-time evaluation would offer more consistent information, especially in the case of nodules with ambiguous features (27). Besides the included US features, nodule stiffness measured through US elastosonography (32) may add value to malignancy risk stratification in this population and should be investigated in high-quality prospective studies. In this meta-analysis, the prevalence of thyroid cancer was relatively high. Although the malignancy rate is overall higher in the pediatric population compared to adults, the risk of pre-selection bias cannot be excluded, as only studies including histology and/or cytology as reference diagnostic tests were considered. Thus, the estimated pre-test probability of malignancy is high, and the US features’ predictive values might not be fully representative of the general population. Finally, most malignant cases are papillary thyroid cancers, so that specific features of less common histotypes in this age group could not be elucidated, as reported for the general population (33).

## Conclusions

This meta-analysis reveals that, in addition to clinical context (i.e. family history, history of exposure to ionizing radiation, childhood cancer survivors), the detection at neck US of suspicious lymph nodes and/or thyroid nodules with a ‘taller than wide’ shape, microcalcifications, and irregular margins are associated with the highest diagnostic accuracy in detecting thyroid cancer in the transition age. These results provide important information for the selection of thyroid nodules candidates for FNA in this setting of patients, limiting the procedure only to cases where it is necessary. Therefore, this could help the physician in patients’ counseling and in tailoring clinical decisions in the transition age. In particular, the suggestion could be that every patient in transition age with a thyroid nodule harboring one of the aforementioned high-risk US features should undergo additional diagnostic evaluation. Conversely, the physician could adopt a conservative approach, deciding for a US follow-up. Future prospective studies are needed to confirm these data.

## Declaration of interest

The authors declare that there is no conflict of interest that could be perceived as prejudicing the impartiality of this work.

## Funding

This work did not receive any specific grant from any funding agency in the public, commercial, or not-for-profit sector.

## Author contribution statement

C D, C P, A C, T F, G G, C V, I S, M G S, and P L selected the issue and researched studies from databases. A C, T F, and G G screened the papers retrieved during the searches. C V, I S, M G S, and P L performed quality control checks on extracted data, performed risk of bias assessment and conceived tables. I S and M M analyzed data. D G, A M I, C P, and C D contributed to the final version of the manuscript. All authors contributed to the article and approved the submitted version.
